# Performance of four different microalgae-based technologies in antibiotics removal under multiple concentrations of antibiotics and strigolactone analogue GR24 administration

**DOI:** 10.1038/s41598-024-67156-w

**Published:** 2024-07-11

**Authors:** Jing Huang, Zhengfang Wang, Chunzhi Zhao, Huayun Yang, Lei Niu

**Affiliations:** 1 School of Mathematics and Statistics, Donghua University, Shanghai, 201620 People’s Republic of China; 2 Suzhou Institute of Trade & Commerce, Suzhou, 215009 People’s Republic of China; 3 School of Ecological Technology & Engineering, Shanghai Institute of Technology, Shanghai, 201400 People’s Republic of China; 4 School of Engineering, Hangzhou Normal University, Hangzhou, 311121 People’s Republic of China

**Keywords:** Antibiotics removal, Endophytic bacteria, Microalgae-bacteria-fungi consortium, Phytohormone, Swine wastewater, Biological techniques, Biotechnology

## Abstract

The formation of symbionts by using different combinations of endophytic bacteria, microalgae, and fungi to purify antibiotics-containing wastewater is an effective and promising biomaterial technology. As it enhances the mixed antibiotics removal performance of the bio-system, this technology is currently extensively studied. Using exogenous supplementation of various low concentrations of the phytohormone strigolactone analogue GR24, the removal of various antibiotics from simulated wastewater was examined. The performances of *Chlorella vulgaris* monoculture, activated sludge–*C. vulgaris*–*Clonostachys rosea*, *Bacillus licheniformis*–*C. vulgaris*–*C. rosea*, and endophytic bacteria (S395-2)–*C. vulgaris*–*C. rosea* co-culture systems were systematically compared. Their removal capacities for tetracycline, oxytetracycline, and chlortetracycline antibiotics from simulated wastewater were assessed. *Chlorella vulgaris*–endophytic bacteria–*C. rosea* co-cultures achieved the best performance under 0.25 mg L^−1^ antibiotics, which could be further enhanced by GR24 supplementation. This result demonstrates that the combination of endophytic bacteria with microalgae and fungi is superior to activated sludge–*B. licheniformis*–microalgae–fungi systems. Exogenous supplementation of GR24 is an effective strategy to improve the performance of antibiotics removal from wastewater.

## Introduction

Microalgae-based technologies are very attractive and effective in the fields of wastewater purification and carbon dioxide neutralization^1–3^. For tetracycline antibiotics removal in particular, microalgae have unique properties, enabling them to deal with these types of antibiotics. The detection rate of tetracycline antibiotics in all types of wastewater, especially pig and livestock wastewater, was high, with concentrations ranging from 0.1 to 1 mg L^−1^. The use of tetracycline (TC), oxytetracycline (OTC) and chlortetracycline (CTC), and among the tetracycline antibiotics was relatively high, and they were the main types of residual tetracycline antibiotics present^3^. The high stability of the above three antibiotics makes them not easily degradable in the aqueous environment. Therefore, the study on the removal of these antibiotics using algae technology is more representative and scientifically significant.

The phytohormone strigolactone stimulates the germination of *Orobanche* seeds and the parasitic weed *Striga*; it also triggers hyphal branching within germinating spores in the arbuscular mycorrhizal fungus *Gigaspora margarita*^4^. Strigolactone and the stress-resistance abscisic acid signaling pathway overlap, suggesting that strigolactone might function as an important node in the phytohormone regulatory network^5^. The enhancing effect of strigolactone analogue GR24 supplementation on the performance of microalgae-based system for the removals of antibiotics, nutrients, and CO_2_ has been reported before^6,7^.

The metabolites of plant endophytic bacteria can stimulate the growth and development of plants and increase the biological stress resistance of host plants^8^. Co-culture of microalgae and endophytic bacteria can increase the nutrient removal efficiency from wastewater^9^. Metal-resistant endophytic bacteria in *Silene vulgaris* tissues have diverse effects on accelerating plant growth and supporting Zn and Cd phytoextraction among non-host plants^10^. Three *Bacillus* species endophytes related to *Thymus vulgaris* (including EGY05, EGY21, and EGY25) significantly foster the development of salt-stressed tomato plants^11^. Additionally, endophytic bacteria, including *Bacillus pumilus* 2–1, *Chryseobacterium indologene* 2–2, and *Acinetobacter johnsonii* 3–1, promote the growth and photosynthesis of the sugar beet (*Beta vulgaris*)^12^.

The main bacteria in activated sludge are mycoplasma-like bacteria and their filamentous fungi. The growth and purification performance of bacterial and microalgal symbionts in activated sludge are important factors for improving the purification process. *Chlorella vulgaris* co-cultured with activated sludge has been used to remove inorganic nutrients including nitrogen and phosphorus as well as organic pollutants^13,14^. *Bacillus licheniformis*, co-cultured with microalgae, can efficiently promote the growth of microalgae^15^. *Bacillus licheniformis* can also degrade poly (lactic acid) and related nanocomposites^16^. *Bacillus licheniformis*–*C. vulgaris* co-culture can efficiently remove ammonium and phosphorus from wastewater, and removal efficiencies of up to 86% and 93% can be obtained, respectively^17^. After 10 days of incubation using a co-culture system (1:3 combination of *C. vulgaris* and *B. licheniformis*), 86.55% of soluble chemical oxygen, 80.28% of total dissolved phosphorus, and 88.95% of soluble chemical oxygen demand were removed from synthetic wastewater^18^. *Chlorella vulgaris* co-cultured with *B. licheniformis* achieved much better nutrient removal performances than the microcystis *aeruginosa*–*B. licheniform* co-culture system. The *C. vulgaris*–*B. licheniformis* co-culture system profoundly contributes to the removal of nutrients, including total nitrogen, ammonium, orthophosphate phosphorus, and chemical oxygen demand, and removal rates of up to 88.82%, 84.98%, 84.87%, and 82.25%, respectively, have been achieved^19^.

Although research associated with the removal of antibiotics from wastewater using microalgae technology for purification is gradually expanding, studies related to the removal effect and mechanism of typical antibiotics from water are rare; moreover, studies using microalgae-bacteria/fungi symbiosis technology under GR24 introduction have not been published to date. There are still a number of scientific issues that need to be addressed, e.g., do symbiont defense mechanisms differ depending on the structural and physicochemical properties of the antibiotic? How does the removal of antibiotics differ between algal symbionts? What are the reasons for these differences? To fill this gap in the literature, in this study, different symbiotic systems based on *C. vulgaris*, *C. rosea*, *B. licheniformis*, and endophytic bacteria (S395-2) were constructed and used to treat synthetic wastewater. The level of GR24 addition on the removal performance of typical antibiotics was assessed, and the induction of GR24 and the mechanism of antibiotic removal were further explored. The conclusions of this study provide a theoretical foundation for the effective use of algal technology in the field of wastewater treatment.

## Methods and materials

### *Chlorella* vulgaris propagation

*Chlorella vulgaris* (FACHB-8) used for this experiment was provided by the Freshwater Algae Culture Center of the China Institute of Aquatic Biology. In reference to the relevant literature, the purchased microalgae were cultured in glass conical flasks with BG-11 medium (See Table S1) prior to experimental treatments^20^. A cool-white LED lamp was used to provide light with a 200 μmol m^−2^ s^−1^ light intensity, under a 12-h/12-h light/dark cycle for the expansion period. The expansion time was 7 d and the expansion temperature was 25 ± 2 °C. During the whole period of expansion, flasks were shaken manually three times per day.

### Activated sludge, *B. licheniformis*, and endophytic bacteria (S395-2) culture

The activated sludge used for the experiment was obtained from nitrifying-denitrifying activated sludge collected in one wastewater treatment plant in Jiaxing City, Zhejiang Province, China. *Bacillus licheniformis* (No. 1.7461) was purchased from the Institute of Microbiology, Chinese Academy of Sciences. The obtained *B. licheniformis* was pre-expanded within Luria Bertani medium at 28 ± 1 °C before use. 0.1 M PBS (prepared in sterile ultrapure water) was added to rinse amplified *B. licheniformis*, followed by three repetitions of centrifugation (8000 × *g* for 10 min) and washing. Then, amplified *B. licheniformis* were used to construct an algal–bacterial co-culture system.

For the extraction and culture of endophytic bacteria (S395-2), *C. vulgaris* culture solution (10 mL) was subjected to centrifugation for 10 min at 8000 r min^−1^ under aseptic conditions. The precipitate obtained by centrifugation was processed by repeated washing and centrifugation under the same conditions. Thereafter, sterile water (0.5 mL) was added and the resultant algal slurry was thorough mixed and ground for 20 min. Then, this diluted solution (100 μL) was added to LB solid medium and incubated for 48 h under 37 ± 1 °C. During this time, the cultured algae were examined with sterile water to assess whether bacteria were present. This test was conducted thrice to choose dominant colonies with antibiotic and staining resistance, which were then subjected to streak purification. Moreover, endophytic bacterium S395-2 was cultivated and subsequently expanded using Luria Bertani medium under 37 ± 1 °C. This culture was used to construct the subsequent algal treatment system.

### *Clonostachys rosea* culture

A *C. rosea* strain single colony was isolated from the fresh soil of Yushu, Qinghai province of China as previously described^21^.

### Construction of four symbiotic algal technologies

The biomass and allocation ratios of microalgae, bacteria, and fungi for all four algal technologies are shown in Table S2.

### Photobioreactor

The photobioreactor used in this study mainly consists of two cylindrical glass tanks (16.8 L) with the same volume. Before the beginning of tests, simulated swine wastewater (2.8 L) was injected into the reactor and purified using different algal treatment techniques. The swine wastewater was added at the glass tank’s wastewater inlet at the right side of the reactor. Later, swine wastewater for purification was gradually pumped into the glass tank from the left side of this reactor using a micro water pump. In the meantime, the inocula of different algae-bacteria/fungus symbionts were supplemented into the left glass tank. The experimental procedure requires that the strains are kept in suspension. To achieve this, a gas distributor was installed at the bottom of the glass tank and air was passed through to prevent the strains from settling. During the experimental treatment, rubber plugs were used to seal both the inlet and sampling ports. Six fluorescent lamps (20 W/110 V) surrounded the left glass tank and provided light throughout the purification process at a light intensity of 200 μmol m^−2^ s^−1^. The reactor was maintained under 25 ± 2 °C and 12-h/12-h light/dark conditions.

### Simulation of wastewater

The utilized simulated swine wastewater contained the following components: MgSO_4_ (2 mg L^−1^), KH_2_PO_4_ (5.62 mg L^−1^), NaH_2_PO_4_.2H_2_O (56.2 mg L^−1^), CaCl_2_ (4 mg L^−1^), urea (120.7 mg L^−1^), glucose (1146.5 mg L^−1^), and antibiotics. In order to examine the removal of tetracycline antibiotics by algal symbiosis, typical CTC, TC, and OTC were selected for treatment. All three antibiotics were purchased from Shanghai McLean Biochemical Co. The final formulated antibiotic-containing culture water had the following relevant indexes: pH 6.92 ± 0.47, chemical oxygen demand 1139.41 ± 53.08 mg L^−1^, total nitrogen 118.63 ± 7.19 mg L^−1^, and total phosphorus 18.73 ± 1.65 mg L^−1^. The simulated antibiotics in the swine wastewater had the same concentrations of the three antibiotics mentioned above, and these were added at the same time. The concentrations were set at four levels of 0.1, 0.25, 0.5, and 1 mg L^−1^, based on two years of antibiotic concentration data from 12 pig farms in Jiaxing, Zhejiang, China.

### Experimental procedure

First, each of the Red: Blue 5:5 (225 μmol m^−2^ s^−1^: 225 μmol m^−2^ s^−1^) was subjected to treatment with four culture modes to optimize conditions. Treatments included Treatment 1 (microalgae *C. vulgaris* monoculture), Treatment 2 (*C. vulgaris*–activated sludge–*C. rosea* co-culture), Treatment 3 (*C. vulgaris*–*B. licheniformis*–*C. rosea* co-culture), and Treatment 4 (*C. vulgaris*–endophytic bacteria (S395-2)–*C. rosea* co-culture).

Initial densities of four treatments were all 119.26 ± 8.54 mg L^−1^. The experimental conditions were 25.0 ± 0.5 °C, white light (intensity, 200 μmol m^−2^ s^−1^), and 12-h/12-h light/dark condition. Next, based on optimal biogas composition and culture conditions, every treatment was carried out in quadruplicates, and average values were obtained, followed by collection of culture samples for analysis at 8:00 am every day.

According to the optimal antibiotics concentration of those four microalgae-based technologies cultured under varying GR24 concentrations (0, 10^−7^, 10^−9^, and 10^−11^ M), Treatment 4 and GR24 concentration with 200 μmol m^−2^ s^−1^ light intensity were chosen as optimal treatment in accordance with the highest antibiotics removal rate. This test was conducted over a 10-day period. All experimental trials in this study are summarized in Table S2.

### Analytical methods and definitions

#### Cell growth measurement

Samples (10 mL) were subjected to centrifugation and washing, followed by filtering via a sterile PES filter membrane (0.45 μm). This membrane (which contained both blank and sample) was processed by 6 h of drying under 105 °C, followed by weighing using an electronic balance. In addition, growth rates (U, d^−1^) under different treatments were determined as follows:

1 $$ {\text{U}} = \left( {{\text{lnDW}}_{{\text{i}}} - {\text{ lnDW}}_{0} } \right)/{\text{t}}_{{\text{i}}} $$

2 $$ {\text{lnDW}}_{{\text{i}}} = {\text{Ut}}_{{\text{i}}} + {\text{lnDW}}_{0} $$

Equation (3) was used to analyze the mean daily productivity (P, g L^−1^ d^−1^):

3 $$ {\text{P}} = \left( {{\text{DW}}_{{\text{i}}} - {\text{DW}}_{0} } \right)/\left( {{\text{t}}_{{\text{i}}} - {\text{t}}_{0} } \right) $$

DW_i_ and DW_0_ (g L^−1^) represent the sample biomass content on day i (t_i_, d) and immediately prior to the experiment (t_0_, d), respectively.

### CHL-a content determination

Strain solution (4 mL) was sampled and centrifuged for 10 min at 8000 r min^−1^. After discarding supernatants, 90% acetone (v/v; 4 mL) was added and the solution was mixed well. After leaving the solution to settle for 24 h under 4 °C in the dark, the sample was subjected to 10 min centrifugation at 8000 r min^−1^. An UV–Vis spectrophotometer was used to detect the absorbance of the supernatant at 630, 645, 663, and 750 nm (measuring OD_663_, OD_750_, OD_645_, and OD_630_, respectively), using 90% acetone as blank control. Chlorophyll a contents (ρ (CHL-a)) under different treatments were determined using Eq. (4):

4 $$ \rho \left( {{\text{CHL}} - {\text{a}}} \right) = {11}.{64} \times \left( {{\text{OD}}_{{{663}}} - {\text{OD}}_{{{75}0}} } \right) + 0.{1}0 \times \left( {{\text{OD}}_{{{63}0}} - {\text{OD}}_{{{75}0}} } \right) - {2}.{16} \times \left( {{\text{OD6}}_{{{45}}} - {\text{OD}}_{{{75}0}} } \right) $$

### Photosynthetic activity analyses

The measurements were conducted using the AquaPen handheld chlorophyll fluorescence meter. Using a chlorophyll fluorescence tester, the fast chlorophyll fluorescence kinetics parameter of samples (4 mL) were measured. The parameters *F*_V_/*F*_M_, *PI*_ABS_, *Φ*_EO_, and *Ψ*_O_ reflect the optimal PSII quantum yield, absorbed light energy-based performance index, electron transfer quantum yield, and electron transfer efficiency because of captured exciton energy, respectively.

### Determination of antibiotics concentration

A water sample (4 mL) was centrifuged for 10 min at 8000 r min^−1^. A 0.45-μm PES filter membrane was then used to filter the supernatant. The concentration of antibiotics was measured using high performance liquid chromatography under the following liquid phase conditions: column: ZORBAX SB-C18 column (5 μm, 250 mm × 4.6 mm); elution mobile phase: acetonitrile: 0.01 M sodium dihydrogen phosphate = 20%: 80%; column temperature, 35 °C; flow rate, 1 mL min^−1^; injection volume, 20 μL; test wavelength, 355 nm. Detection wavelengths for OTC, TC, and CTC were 264 nm, 360 nm, and 319 nm, respectively, and pure water was used as control. Then, antibiotics removal (R) was determined using Eq. (5):

5 $$ {\text{R}} = \left( {{\text{C}}_{0} - {\text{C}}_{{\text{i}}} } \right)/{\text{C}}_{0} \times {1}00 $$

where C_0_ and C_i_ represent the antibiotics concentration (mg L^−1^) prior to and following purification, respectively.

### Statistical analyses

The results are presented as mean ± standard deviation (SD) of three independent assays. Experimental data were processed and statistically analyzed with SPSS19.0 software. Differences among experimental results under different treatment conditions were analyzed by Duncan’s multiple comparison method. *P* < 0.05 was defined as indicating significant differences.

## Results

### Growth under four treatments of various antibiotics concentrations

**Figure 1:**
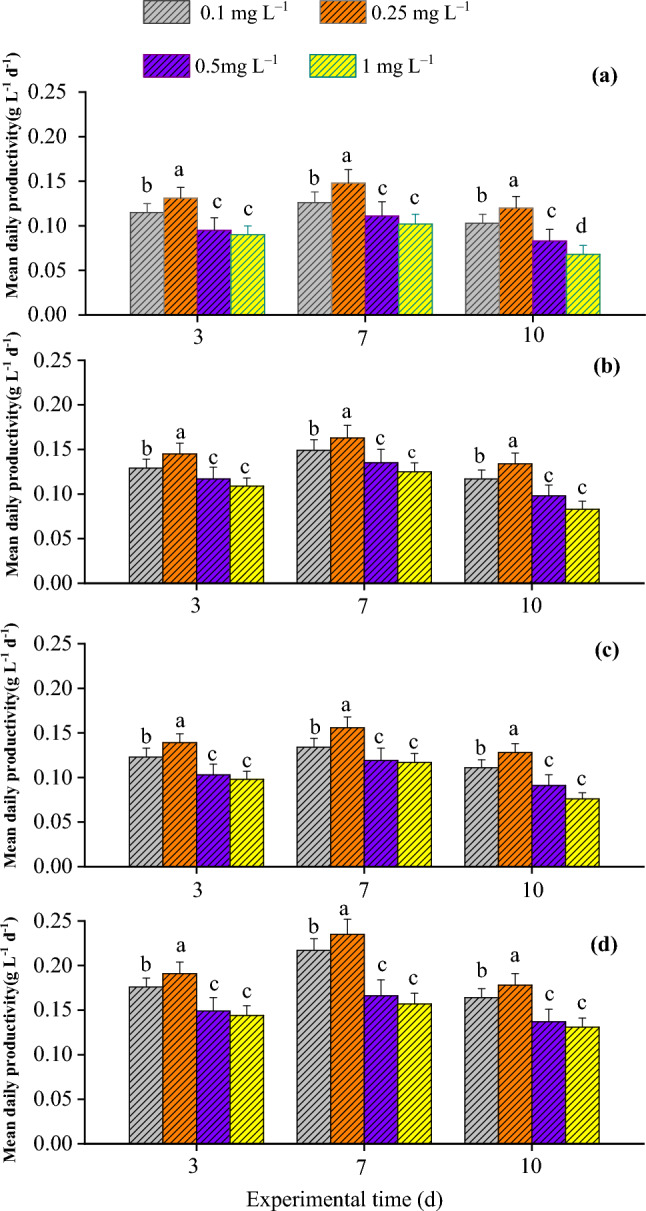
Mean daily productivity of four treatments under various antibiotics concentrations at 3 d, 7 d, and 10 d. (**a**) Treatment 1, (**b**) Treatment 2, (**c**) Treatment 3, and (**d**) Treatment 4.

**Figure 2:**
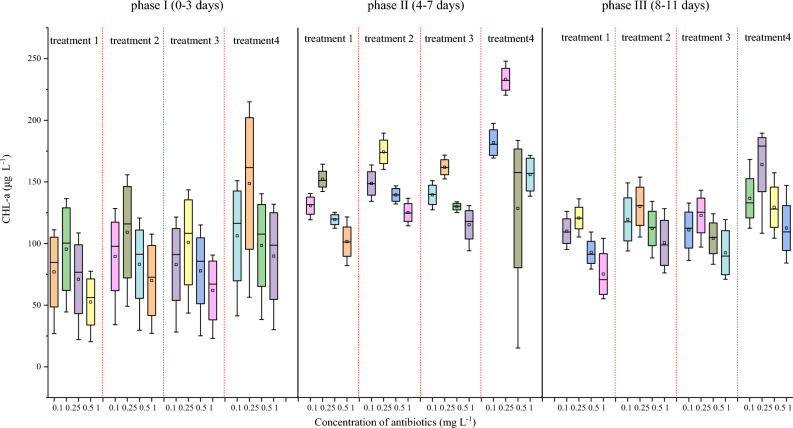
Chlorophyll a contents of the four treatments under various antibiotics concentrations at 0–3 d, 4–7 d, and 8–11 d.

To characterize the effects of various concentrations of antibiotics on four treatments, the contents of CHL-a, daily production, and antibiotics removal efficiency were systematically analyzed. The results showed that *C. vulgaris* monoculture (Treatment 1) achieved the highest CHL-a content and daily production in the presence of 0.25 mg L^−1^ antibiotics at day 3 (Figs. 1a and 2a). At day 7, *C. vulgaris* had higher CHL-a content and daily production under 0.25 mg L^−1^, compared with that at day 3 (Figs. 1a and 2a). At day 10, the CHL-a content and daily production had decreased, compared with those at days 3 and 7 (Figs. 1a, 2a). These results are related to the nutrient status, antibiotics concentration, and the growth characteristics of *C. vulgaris* over time^22,23^.

Treatment 2 (*C. vulgaris*–activated sludge–*C. rosea*) had the highest CHL-a content and daily production at day 3 under 0.25 mg L^−1^ antibiotics (Figs. 1b and 2b). As incubation time increased to 7 days, the CHL-a content and daily production of Treatment 2 further increased under 0.25 mg L^−1^ antibiotics treatment, which dropped at day10 (Figs. 1b and 2b). Treatment 3 (microalgae–*B. licheniformis*–*C. rosea*) at day 3 and under 0.25 mg L^−1^ antibiotics had peak CHL-a content and daily production, which decreased at day 10 (Figs. 1c and 2c). Treatment 4 (*C. vulgaris*–endophytic bacteria (S395-2)–*C. rosea*) had the highest CHL-a content and daily production at day 7 under 0.25 mg L^−1^ antibiotics, which decreased at day 10 (Figs. 1d and 2d). Among the four systems tested, Treatment 4 was the best combination, as it achieved the highest CHL-a content and daily production at day 3 (Fig. 1a–d). These results demonstrate that 0.25 mg L^−1^ antibiotics condition was optimal for maximal CHL-a content and daily production under all four-biomaterial systems.

Treatment 4 had the highest CHL-a contents and daily production at day 7. The time to reach the growth plateau will vary for different dominant algal strains, which is mainly related to the microalgae species, the choice of medium and the characteristics of wastewater constituents^24^. The density level of the strains gradually increased with incubation time, reached the extreme value to enter the plateau period, and the daily production decreased at the end stage of growth. The removal efficiency of the target antibiotics was basically consistent with the trend of strains density^25^. The growth of the strains reached the optimal state in the 7th d, which may indicate that the biosorption in the antibiotic removal process has reached saturation at this time, but the biodegradation process includes both intracellular and extracellular pathways, which are related to the structure of microbial community in the wastewater, real-time concentration of antibiotics, concentration of nutrient salts, and structure of the reactor, and characteristics of the wastewater influent. Therefore, the removal of the target antibiotics was carried out throughout the experimental period, only that its removal rate would be changed by the above factors.

*Chlorella vulgaris* monoculture under 0.5 and 1 mg L^−1^ antibiotics treatments had lower daily production compared with that under 0.1 mg L^−1^ antibiotic condition. This result is consistent with the notion that antibiotics are toxic to microalgae and inhibit their growth by suppressing chloroplast formation, chlorophyll production, protein synthesis, and gene expression^26–28^. Interestingly, 0.25 mg L^−1^ antibiotics had significantly higher daily production compared with 0.1 mg L^−1^ antibiotics. This result suggests that 0.25 mg L^−1^ antibiotics induced the appropriate toxicity level, maximal adaptability, stress tolerance, and cell metabolism adjustment so that *C. vulgaris* reached the highest daily production compared with relatively low antibiotics concentrations. These results are slightly different from the data showing that < 50 mg L^−1^ OTC promotes the development of microalgae, while > 100 mg L^−1^ OTC suppresses their development^29^. *Chlorella vulgaris* and three bacteria*–C.vulgaris*–*C. rosea* symbionts all had similar daily production changing trends following the four different concentrations of antibiotics (i.e., 0.1, 0.25, 0.5, and 1.0 mg L^−1^). These findings demonstrate that the antibiotics tolerance of *C. vulgaris* monoculture is lower than that of the other three bacteria–*C. vulgaris*–*C. rosea* symbionts (Fig. 1). Furthermore, the endophytic bacteria (S395-2)–*C. vulgaris*–*C. rosea* symbiont had the most prominent daily production performance under 0.25 mg L^−1^ antibiotics. These findings are consistent with the mechanism through which endophytic bacteria help microalgae to change their binding mode to remove antibiotics^30^.

### Photosynthetic abilities under four treatments at different antibiotics concentrations

The *F*_V_/*F*_M_ ratio value marks chlorophyll I fluorescence of PSII, which is an indicator of PSII activity and usually used to identify environmental stress^31^. Table 1 lists the photosynthetic performance of all four treatments at day 10. Among all four treatments, Treatment 2 had the best* F*_V_/*F*_M_ performance under 0.25 mg L^−1^ antibiotics, and Treatment 4 had the highest *F*_V_/*F*_M_ performance at 0.95 ± 0.09 (Table 1). For *C. vulgaris* and three different bacteria–*C. vulgaris–C. rosea* symbionts, 0.25 mg L^−1^ antibiotics treatment increased the *F*_V_/*F*_M_ value compared with 0.1 mg L^−1^ antibiotics. Increases were from 0.51 ± 0.05 to 0.72 ± 0.07 for Treatment 1, from 0.63 ± 0.06 to 0.79 ± 0.08 for Treatment 2, from 0.73 ± 0.07 to 0.86 ± 0.09 for Treatment 3, and from 0.83 ± 0.08 to 0.95 ± 0.09 for Treatment 4 (Table 1).

**Table 1:** Fluorescence data obtained via fast chlorophyll fluorescence kinetics test on the 10th day for all four selected fungi-assisted microalgal treatments.

	Treatment 1	Treatment 2	Treatment 3	Treatment 4
Antibiotics concentrations	*F*V/*F*M			
0.1 mg L^−1^	0.51^b^ ± 0.05	0.63^b^ ± 0.06	0.73^b^ ± 0.07	0.83^b^ ± 0.08
0.25 mg L^−1^	0.72^a^ ± 0.07	0.79^a^ ± 0.08	0.86^a^ ± 0.09	0.95^a^ ± 0.09
0.5 mg L^−1^	0.48^b^ ± 0.05	0.56^b^ ± 0.06	0.51^c^ ± 0.05	0.64^c^ ± 0.06
1 mg L^−1^	0.36^c^ ± 0.03	0.49^c^ ± 0.05	0.47^c^ ± 0.05	0.56^c^ ± 0.06
	*PI*ABS			
0.1 mg L^−1^	4.73^b^ ± 0.41	4.98^b^ ± 0.46	5.93^b^ ± 0.57	6.63^b^ ± 0.62
0.25 mg L^−1^	5.42^a^ ± 0.50	5.92^a^ ± 0.57	6.76^a^ ± 0.64	7.58^a^ ± 0.71
0.5 mg L^−1^	4.36^b^ ± 0.42	4.84^b^ ± 0.45	5.43^b^ ± 0.51	6.17^b^ ± 0.59
1 mg L^−1^	3.94^c^ ± 0.36	4.12^c^ ± 0.39	4.82^c^ ± 0.45	5.22^c^ ± 0.48
	*Ψ*O			
0.1 mg L^−1^	0.74^a^ ± 0.07	0.84^a^ ± 0.08	0.92^a^ ± 0.09	0.92^a^ ± 0.09
0.25 mg L^−1^	0.83^a^ ± 0.08	0.87^a^ ± 0.09	0.95^a^ ± 0.09	0.96^a^ ± 0.09
0.5 mg L^−1^	0.61^b^ ± 0.06	0.64^b^ ± 0.06	0.79^b^ ± 0.08	0.81^b^ ± 0.08
1 mg L^−1^	0.57^b^ ± 0.06	0.61^b^ ± 0.06	0.76^b^ ± 0.08	0.77^b^ ± 0.08
	*Φ*EO			
0.1 mg L^−1^	0.51^a^ ± 0.05	0.58^a^ ± 0.06	0.67^a^ ± 0.07	0.69^a^ ± 0.07
0.25 mg L^−1^	0.54^a^ ± 0.05	0.63^a^ ± 0.06	0.73^a^ ± 0.07	0.73^a^ ± 0.07
0.5 mg L^−1^	0.36^b^ ± 0.03	0.41^b^ ± 0.04	0.51^b^ ± 0.05	0.56^b^ ± 0.06
1 mg L^−1^	0.32^b^ ± 0.03	0.36^b^ ± 0.04	0.48^b^ ± 0.05	0.54^b^ ± 0.05

The data in this Table are expressed as mean ± SD (n = 3). Values with different superscript letters indicate a significant difference at *P* < 0.05.

These results are consistent with the daily production data, further showing that 0.25 mg L^−1^ antibiotics efficiently increased growth compared with 0.1 mg L^−1^ antibiotics (Table 2). However, among the four treatments, 0.5 and 1 mg L^−1^ antibiotics treatment decreased *F*_V_/*F*_M_ ratios compared with 0.25 mg L^−1^ antibiotics treatment (Table 1). The decrease of *F*_V_/*F*_M_ values can be attributed to the inactivation of the PSII reaction center of microalgal cells. This suggests that higher antibiotics concentrations are toxic and can inhibit photosynthesis-related protein synthesis and gene expression such as PSII reaction centers. These results are consistent with the inhibitory effects of antibiotics toxicity on microalgal growth^26^.

**Table 2:** Growth rates and mean daily productivity levels of the four selected strains under various antibiotics concentration treatments.

	Treatment 1	Treatment 2	Treatment 3	Treatment 4
Antibiotics concentrations	Growth rate d^−1^			
0.1 mg L^−1^	0.191^b^ ± 0.02	0.281^b^ ± 0.03	0.238^b^ ± 0.02	0.302^b^ ± 0.03
0.25 mg L^−1^	0.217^a^ ± 0.02	0.319^a^ ± 0.03	0.281^a^ ± 0.03	0.337^a^ ± 0.04
0.5 mg L^−1^	0.182^b^ ± 0.02	0.262^b^ ± 0.03	0.223^b^ ± 0.02	0.283^c^ ± 0.03
1 mg L^−1^	0.143^c^ ± 0.01	0.213^c^ ± 0.02	0.179^c^ ± 0.02	0.235^d^ ± 0.02
	Mean daily productivity (g L^−1^ d^−1^)			
0.1 mg L^−1^	0.117^b^ ± 0.010	0.141^b^ ± 0.014	0.128^b^ ± 0.012	0.159^b^ ± 0.015
0.25 mg L^−1^	0.134^a^ ± 0.011	0.166^a^ ± 0.015	0.147^a^ ± 0.013	0.182^a^ ± 0.018
0.5 mg L^−1^	0.109^b^ ± 0.010	0.134^b^ ± 0.013	0.121^b^ ± 0.011	0.146^c^ ± 0.014
1 mg L^−1^	0.094^c^ ± 0.009	0.126^c^ ± 0.011	0.113^c^ ± 0.010	0.133^d^ ± 0.013

The data in this table are expressed as mean ± SD (n = 3). Values with different superscript letters indicate a significant difference at *P* < 0.05.

Higher concentrations (0.5 and 1 mg L^−1^ of antibiotics compared with 0.25 mg L^−1^) imposed a significant inhibitory effect on the photosynthetic activity of all four treatments tested. The absorption performance index of light energy (*PI*_ABS_) decreased significantly under all four treatments (Table 1). Consistent with a reduction of electron transfer efficiency (*Ψ*_O_) and electron transfer quantum yield (*Φ*_EO_), a reduction in the maximum optical efficiency (*F*_V_/*F*_M_) was observed under all four treatments under 0.5 and 1 mg L^−1^ antibiotics concentrations (Table 1). All four treatments had their highest values under 0.25 mg L^−1^ antibiotics condition, where Treatment 4 had the highest value with *PI*_ABS_ 7.58 ± 0.71 (Table 1). For *Ψ*_O_ to mark light absorption, all four treatments had their highest value under 0.25 mg L^−1^ antibiotics condition, and Treatment 4 had the highest value at 0.96 ± 0.09 (Table 1). The *Φ*_EO_ parameters, which reflects photosynthetic activity, all reached the highest level under 0.25 mg L^−1^ antibiotics and Treatment 4 achieved the highest level of 0.73 ± 0.07 (Table 1). Based on these results, 0.25 mg L^−1^ antibiotics concentration represents the best concentration for *C. vulgaris* monoculture and three different bacteria–*C. vulgaris*–*C. rosea* co-culture systems to achieve the best photosynthetic performance. Among all treatments tested, Treatment 4 achieved the best results.

### Antibiotics removal efficiencies under various antibiotics concentration of the four selected strains

To further characterize Treatment 4, various concentrations of the antibiotics OTC, TC, and CTC were used and removal efficiencies were systematically determined. Compared to Treatment 1, under various antibiotics concentrations, Treatments 2, 3, and 4 all had significantly higher antibiotics removal efficiencies (Table 3). These results are consistent with the growth and photosynthetic activity data, i.e., three different bacteria–*C. vulgaris*–*C. rosea* mixtures achieved better performances than *C. vulgaris* monoculture.

**Table 3:** Mean values ± SD of the removal efficiencies of CTC, TC, and OTC for all four selected microalgae-based technologies under various antibiotics concentrations.

	Treatment 1	Treatment 2	Treatment 3	Treatment 4
Antibiotics concentrations	CTC removal efficiency (%)			
0.1 mg L^−1^	91.85^a^ ± 6.26	95.83^ab^ ± 5.02	94.19^ab^ ± 4.03	96.17^ab^ ± 2.06
0.25 mg L^−1^	93.74^a^ ± 6.31	97.72^a^ ± 5.56	96.54^a^ ± 2.18	98.61^a^ ± 1.39
0.5 mg L^−1^	86.49^b^ ± 7.15	89.35^bc^ ± 5.13	88.53^bc^ ± 5.12	92.42^bc^ ± 4.55
1 mg L^−1^	82.93^b^ ± 7.62	86.71^c^ ± 5.27	85.06^c^ ± 5.37	87.53^c^ ± 4.92
	TC removal efficiency (%)			
0.1 mg L^−1^	82.55^b^ ± 6.25	87.45^ab^ ± 7.39	84.36^a^ ± 7.78	89.83^b^ ± 7.05
0.25 mg L^−1^	85.16^a^ ± 7.02	89.68^a^ ± 7.53	86.53^a^ ± 7.61	94.19^a^ ± 5.04
0.5 mg L^−1^	78.91^bc^ ± 5.93	78.92^c^ ± 7.15	77.06^b^ ± 7.18	83.61^c^ ± 6.93
1 mg L^−1^	74.59^c^ ± 5.57	76.28^c^ ± 6.91	74.37^b^ ± 6.52	80.98^c^ ± 6.77
	OTC removal efficiency (%)			
0.1 mg L^−1^	89.15^ab^ ± 8.62	92.74^ab^ ± 5.69	90.28^ab^ ± 6.35	94.81^ab^ ± 4.75
0.25 mg L^−1^	92.35^a^ ± 5.73	95.88^a^ ± 2.91	93.76^a^ ± 6.07	98.47^a^ ± 1.03
0.5 mg L^−1^	84.69^b^ ± 7.76	88.39^bc^ ± 6.52	86.03^b^ ± 5.84	91.98^b^ ± 4.36
1 mg L^−1^	80.86^c^ ± 7.53	83.67^c^ ± 6.34	80.27^c^ ± 5.16	86.79^c^ ± 6.24

Values with different superscript letters indicate significant differences at *P* < 0.05 according to Duncan’s multiple range tests for the same strain under different treatments.

**Figure 3:**
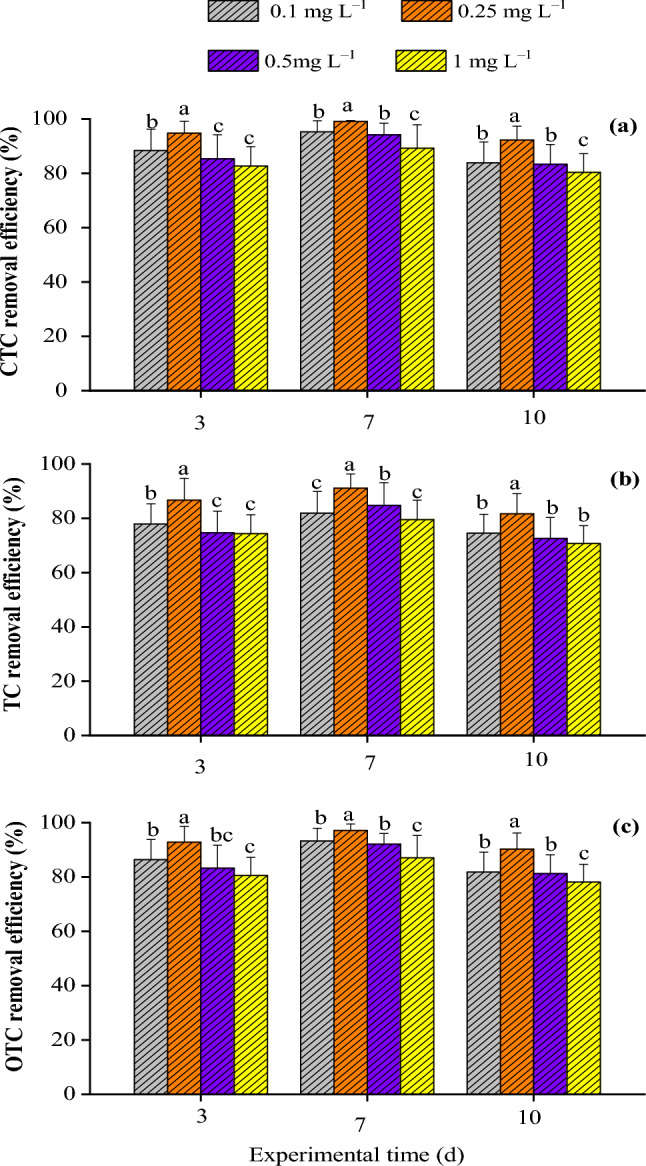
Antibiotics removal efficiencies of Treatment 4 under various antibiotics concentrations at 3 d, 7 d, and 10 d. (**a**) CTC removal efficiency, (**b**) TC removal efficiency, and (**c**) OTC removal efficiency. *Note*: The same letters indicate a non-significant difference in data (*p* > 0.05) and different letters indicate a significant difference (*p* < 0.05).

The growth of strains was divided into three stages, namely the early stage of growth (0–3 d), the growth bloom (4–7 d), and the end of growth (8–11 d). The effectiveness regarding the removal antibiotics was closely related to the growth characteristics of strains, which mainly depend on the biological fixation process. Treatment 4 had the highest CTC removal efficiency of up to 99.09 ± 0.36 under 0.25 mg L^−1^ antibiotics at day 7, compared with those on days 3 and 10 (Fig. 3a). The TC removal efficiency of Treatment 4 reached 91.08 ± 5.24 at day 7 under 0.25 mg L^−1^, which was much higher than removal efficiencies at days 3 and 10, with values of 86.67 ± 8.01 and 81.62 ± 7.74, respectively (Fig. 3b). Moreover, Treatment 4 also had the highest OTC removal efficiency under 0.25 mg L^−1^ antibiotics with a value of up to 97.11 ± 2.43 at day 7. This was higher than values at days 3 and 10, at 92.8 ± 5.88 and 90.28 ± 5.95, respectively (Fig. 3c).

Table 3 shows the average value of three kinds of antibiotics-containing simulated wastewater purifications using four different treatments under various antibiotics concentrations during a 10-day experimental process. The results show that Treatment 4 achieved the highest CTC removal efficiency under four different antibiotics treatments, where 0.25 mg L^−1^ CTC achieved best removal efficiency with a value of up to 98.61 ± 1.39% (Table 3). Treatment 4 also achieved the best removal performance for TC under the 0.25 mg L^−1^ condition, with a value of up to 94.19 ± 5.04% (Table 3). Under the 0.25 mg L^−1^ concentration, Treatment 4 also achieved the highest OTC removal with a value of up to 98.47 ± 1.03% (Table 3). The treatment removal efficiencies can be ranked as CTC > OTC > TC.

The removal efficient of the target antibiotics was correlated with the N and P contents in the wastewater. Zhou et al.^32^ found that *Chlorella proteus* had an enhancement effect on the removal of cephradine at high nitrogen and high phosphorus levels, and its removal rate of the target antibiotics reached more than 90% at 3 d. In the whole process, the synergistic effect of N and P gradually weakened with the time of reaction, and the main synergistic effect occurred in the period of 6–24 h. The difference in the content of nitrogen and phosphorus did not have a significant synergistic effect on the removal ability of *Chlorella*, while the effect of high content of nitrogen and phosphorus nutrients was more obvious in the promotion of the growth ability of microalgae (population growth) and the photosynthesis ability (content of photosynthetic pigments). In this study, the starting TN and TP levels (118.63 ± 7.19 mg L^−1^ and 18.73 ± 1.65 mg L^−1^, respectively) were relatively high, which had a certain synergistic effect on the removal of target antibiotics. However, in the practical application of antibiotic removal from wastewater, due to the complex composition of wastewater and the variety of elemental forms, the specific effects and removal mechanisms need to be further studied.

### Antibiotics removal efficiency under various GR24 concentrations for Treatment 4

The effect of variations in exogenous additions (0, 10^−11^, 10^−9^, and 10^−7^ M) of GR24 on antibiotics removal were examined for all four algal treatment techniques. The concentration of all three antibiotics (CTC, TC, and OTC) in the simulated swine wastewater were 0.25 mg L^−1^. Table 4 shows the antibiotic removal efficiencies after 10 d of purification.

**Table 4:** Mean values ± SD of the removal efficiencies of CTC, TC, and OTC at 0.25 mg L^−1^ for antibiotics under various GR24 concentrations.

	Treatment 1	Treatment 2	Treatment 3	Treatment 4
GR24 concentrations	CTC removal efficiency (%)			
0 M	93.74^a^ ± 6.31	97.72^a^ ± 5.56	96.54^a^ ± 2.18	98.61^a^ ± 1.39
10^−7^ M	94.63^a^ ± 6.52	97.91^a^ ± 5.89	97.25^a^ ± 2.35	98.97^a^ ± 1.06
10^−9^ M	96.33^a^ ± 3.04	98.54^a^ ± 1.16	98.03^a^ ± 1.67	99.27^a^ ± 0.25
10^−11^ M	94.09^a^ ± 5.13	97.38^a^ ± 6.27	96.98^a^ ± 3.15	98.74^a^ ± 1.03
	TC removal efficiency (%)			
0 M	82.55^c^ ± 6.25	87.45^c^ ± 7.39	84.36^c^ ± 7.78	89.83^c^ ± 7.05
10^−7^ M	87.06^b^ ± 6.91	92.31^b^ ± 6.05	89.52^b^ ± 7.93	94.76^b^ ± 5.11
10^−9^ M	92.87^a^ ± 7.01	97.17^a^ ± 2.04	94.17^a^ ± 5.21	99.24^a^ ± 0.32
10^−11^ M	86.83^b^ ± 6.77	90.59^bc^ ± 5.71	87.26^c^ ± 6.95	92.57^bc^ ± 4.83
	OTC removal efficiency (%)			
0 M	92.35^a^ ± 5.73	95.88^a^ ± 2.91	93.76^a^ ± 6.07	98.47^a^ ± 1.03
10^−7^ M	93.86^a^ ± 5.91	96.52^a^ ± 3.25	95.05^a^ ± 4.32	98.92^a^ ± 0.74
10^−9^ M	95.72^a^ ± 4.14	97.61^a^ ± 2.03	96.34^a^ ± 3.28	99.18^a^ ± 0.57
10^−11^ M	93.09^a^ ± 5.86	96.24^a^ ± 2.26	94.81^a^ ± 4.39	98.23^a^ ± 1.05

Values with different superscript letters indicate a significant difference at *P* < 0.05 according to Duncan’s multiple range tests for the same strain under different treatments.

As shown in Table 4, the impact of GR24 addition levels on the removal of three antibiotics was similar in all four treatment systems. The change of GR24 addition level had no effect on the removal rate of both CTC and OTC. A GR24 addition of more than 10^−11^ M improved the TC removal rate to a certain extent. With increasing concentration level of GR24, the TC removal rate exhibited a trend of first increasing followed by a decrease. The best TC removal rates were achieved under four treatments at GR24 concentration levels of 10^−9^ M. Removal rates were 92.87 ± 7.01% (Treatment 1), 97.17 ± 2.04% (Treatment 2), 94.17 ± 5.21% (Treatment 3), and 99.24 ± 0.32% (Treatment 4). While all four treatments exhibited similar trends for the removal of the three antibiotics, their removal rates can be ordered as follows: Treatment 4 > Treatment 2 > Treatment 3 > Treatment 1.

## Discussion

### Analysis of the induction of GR24

The GR24 enhancement of antibiotics tolerance in the tested strains generally used a 2-tier mechanism: (1) Exogenous phytohormones can enhance the tolerance of microalgae to abiotic stresses by activating the antioxidant system. Under abiotic stresses, phytohormone-induced production of additional non-enzymatic antioxidants leads to a more direct scavenging of intracellular reactive oxygen species (ROS)^33^. Phytohormone-induced production of additional polyunsaturated fatty acids or carotenoids, among others, contributes to mitigating the effects of the interaction between ROS and their double bonds. In addition, phytohormones can act as natural antioxidants, thus directly scavenging excessive ROS^34^. (2) GR24 can enhance the resilience of microalgae by modulating their signaling network^35^. The signaling network of microalgae is very complex; in addition to stress hormones, phytohormone application can also regulate the levels of other endogenous phytohormones. These interact with each other in a complex network, which enables them to respond synergistically or antagonistically to exogenous environmental stressors^36^. The results of the present study corroborate that GR24 plays a key role in increasing the activities of various antioxidant enzymes as well as in modulating the signaling network of microalgae.

### Analysis of the action mechanism of antibiotics removal

In microalgae-based systems, the growth of the strains directly affected the removal efficiency of the target antibiotics^37^. Antibiotics can be eliminated both intracellularly and extracellularly through many biological processes such as biodegradation, bioaccumulation, absorption, photolysis, and hydrolysis^26^. It has been shown that bacteria–microalgae–fungi symbioses changes the binding mode from adhesive to fusogenic and produces more extracellular polymeric substances (EPS); these effects positively contribute to the removal of antibiotics^30^. This is one of the important reasons why the antibiotic removal performance of the algal–bacterial–fugal three-phase symbiosis outperforms that of two-phase symbiosis (algal–bacterial and algal–fugal) compared to microalgae monoculture. When antibiotics come into contact with algal symbionts, antibiotic-like substances are adsorbed by the cell walls and EPS of microalgae^38^. A prior study confirmed the protective role of microalgal EPS in the resistance to antibiotic stress^39^. Bioaccumulation is mainly caused by the ability of microalgae in algal symbioses to take up antibiotics from the external environment and bind them to microalgal intracellular proteins or other compounds^40^. A related study showed that antibiotics can cross the cell membrane of microalgae and enter the cell through passive diffusion, passive facilitated diffusion, and active uptake^41^.

Biodegradation of antibiotics depends on the cellular metabolism of microalgae. It involves oxidation, reduction, and hydrolysis reactions at the first stage^42^ and in the second stage process, the products of the first stage bind to polar substances such as sugars and amino acids^43^. The biodegradation of sulfamethoxazole by *C. vulgaris* involves the first stage of oxidation of amine groups under enzymatic catalysis and a two-stage reaction process of hydroxylation and formylation^44^. As reason for the enhanced antibiotic removal efficiency of algal symbiosis, the mutually beneficial symbiotic relationship between *C. vulgaris* and mycelium was suggested^3^. However, fungal hyphae can protect microalgae by extracellular polysaccharide adhesion, surface protein interactions, and electrostatic neutralization, thus preventing the microalgae from being damaged by antibiotics or other pollutants^45^. Flowchart of antibiotic removal mechanisms by co-cultures are shown in Fig S1.

### Feasible application in the future

Algae-bacteria-fungal symbiosis purification technology has a wide range of applications in the future^3,24^. For example, it can be designed as a box in which suitable concentration of algal and bacterial/fungal symbiosis is placed. The box can be moved freely and repeatedly within the treated waters by remote control to achieve biological purification of water in ponds or wastewater areas. The treated algal organisms can be used as biofuel, anaerobic fermentation, diesel production, etc^23,41^. For wastewater with different antibiotic contamination characteristics, the best algal technology is selected to maximize the reduction of pollutants, taking into account factors such as removal effect and economy.

## Conclusions

The co-culture endophytic bacteria (S395-2)–*C. vulgaris*–*C. rosea* application was found to achieve superior antibiotics removal compared to three other algal treatment technologies. GR24 further enhanced the antibiotics removal performance of this system. These findings suggest that the endophytic bacteria (S395-2)–*C. vulgaris*–*C. rosea* co-culture system had the best antibiotics stress-resistance, adaptability, growth, and biomass production in simulated wastewater after a total of 10 d of incubation. Under this scenario, GR24 supplementation further enhanced the antibiotics removal efficiency, CHL-a content, and daily production. Future work should conduct large-scale tests and use actual wastewater to further optimize the GR24-treated endophytic bacteria (S395-2)–*C. vulgaris*–*C. rosea* co-culture system for application in actual antibiotics wastewater treatment.

### Supplementary Information

Supplementary Information.

## Data Availability

All data generated or analysed during this study are included in this published article [and its Supplementary information files].
