# Supplementary material for: Performance of four different microalgae-based technologies in antibiotics removal under multiple concentrations of antibiotics and strigolactone analogue GR24 administration
Source: Sci Rep. 2024 Jul 11;14:16004. doi: 10.1038/s41598-024-67156-w (PMC11239813; doi:10.1038/s41598-024-67156-w)

**Table S1**  The composition of BG-11 medium

| Components | Values |
| --- | --- |
| EDTA | 0.001 g L^-1^ |
| Ferric ammonium citrate | 0.006 g L^-1^ |
| Citric acid | 0.006 g L^-1^ |
| CaCl_2_·2H_2_O | 0.036 g L^-1^ |
| K_2_HPO_4_·3H_2_O | 0.04 g L^-1^ |
| Na_2_CO_3_ | 0.02 g L^-1^ |
| MgSO_4_·7H_2_O | 0.075 g L^-1^ |
| NaNO_3_ | 1.5 g L^-1^ |
| 1.0 mL trace metal mixture A_5_ (pH=7.1)  CoCl_2_·6H_2_O  ZnSO_4_·7H_2_O  H_3_BO_3_  NaMoO_4_·2H_2_O  CuSO_4_·5H_2_O  MnCl_2_·4H_2_O | 0.05 g L^-1^  0.222 g L^-1^  2.86 g L^-1^  0.39 g L^-1^  0.079 g L^-1^  1.81 g L^-1^ |

**Table S2** All experimental trials in this study

| Treatment  types | Microorganism | | | Concentration gradient of antibiotics (mg L^-1^) | | | | Concentration of GR24 (M) | | | |
| --- | --- | --- | --- | --- | --- | --- | --- | --- | --- | --- | --- |
|  | Microalgae | Bacteria | Fungi | 0.05 | 0.1 | 0.25 | 1 | 0 | 10^-7^ | 10^-9^ | 10^-11^ |
| Treatment 1 | *C.vulgaris* ( 2.0×10^6^ cells mL^-1^) | / | / | √ | √ | √ | √ | √ | √ | √ | √ |
| Treatment 2 | *C.vulgaris* (2.0×10^6^ cells mL^-1^) | Activated sludge (2.0×10^7^ cell mL^-1^) | *C. Rosea* (1.0×10^6^ spores mL^-1^) | √ | √ | √ | √ | √ | √ | √ | √ |
| Treatment 3 | *C.vulgaris* (2.0×10^6^ cells mL^-1^) | *B.licheniformis* (2.0×10^7^ cell mL^-1^) | *C. Rosea* (1.0×10^6^ spores mL^-1^) | √ | √ | √ | √ | √ | √ | √ | √ |
| Treatment 4 | *C.vulgaris* (2.0×10^6^ cells mL^-1^) | S395-2 (2.0×10^7^ cell mL^-1^) | *C. Rosea* (1.0×10^6^ spores mL^-1^) | √ | √ | √ | √ | √ | √ | √ | √ |

**Fig S1** Flowchart of antibiotic removal mechanisms by co-cultures.


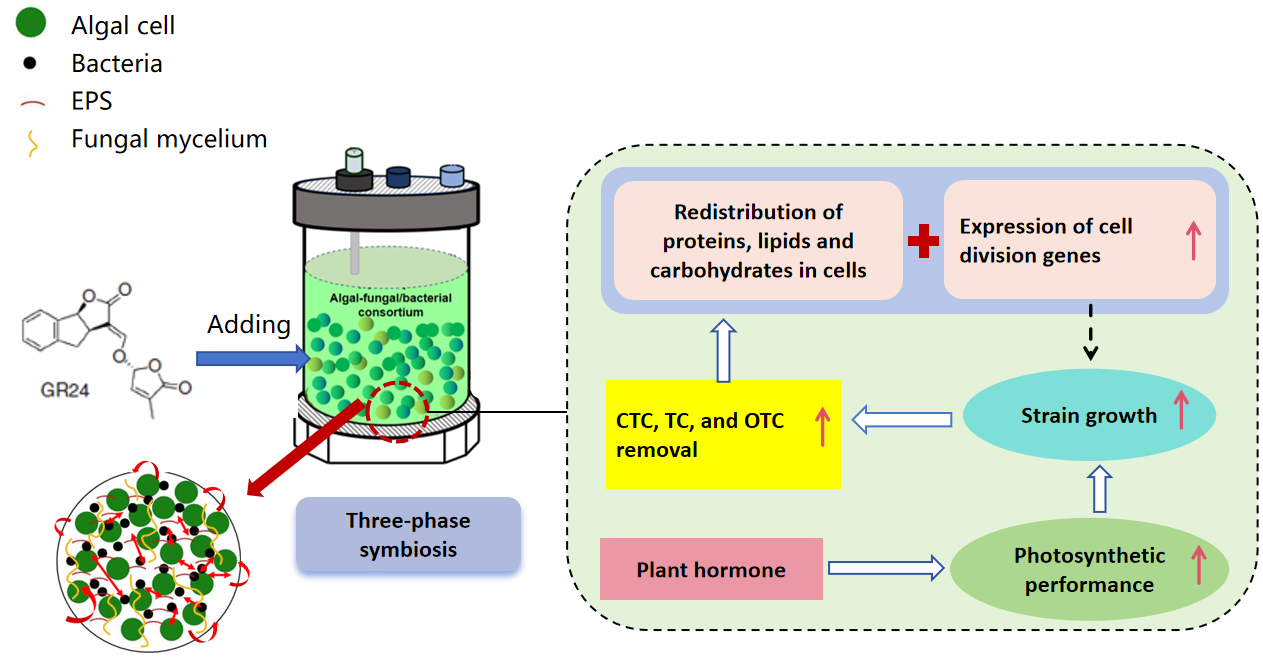

Supplement: Supplementary file 1 — Supplementary Information. [file 41598_2024_67156_MOESM1_ESM.docx]
